# Capturing omega sign in the clinical assessment of depression by deep learning

**DOI:** 10.1038/s41398-026-04245-6

**Published:** 2026-07-07

**Authors:** Jie Chen, Chun-Tung Li, Ngan Yin Chan, Chris Xie Chen, Sijing Chen, Joey W. Y. Chan, Yaping Liu, Shirley Xin Li, Steven W. H. Chau, Kwong Sak Leung, Pheng-Ann Heng, Tatia M. C. Lee, Tim M. H. Li, Yun-Kwok Wing

**Affiliations:** 1 Li Chiu Kong Family Sleep Assessment Unit, Department of Psychiatry, Faculty of Medicine, The Chinese University of Hong Kong, Shatin Hong Kong SAR, China; 2 Department of Psychiatry, Fuzhou Neuropsychiatric Hospital Affiliated to Fujian Medical University, Fuzhou, China; 3 Center for Sleep and Circadian Medicine, The Affiliated Brain Hospital of Guangzhou Medical University, Guangzhou, Guangdong China; 4 State Key Laboratory of Brain and Cognitive Sciences, The University of Hong Kong, Hong Kong SAR, China; 5 Sleep Research Clinic and Laboratory, Department of Psychology, The University of Hong Kong, Hong Kong SAR, China; 6 Department of Computer Science and Engineering, The Chinese University of Hong Kong, Hong Kong SAR, China; 7 Laboratory of Neuropsychology and Human Neuroscience, Department of Psychology, The University of Hong Kong, Hong Kong SAR, China; 8 Li Ka Shing Institute of Health Sciences, Faculty of Medicine, The Chinese University of Hong Kong, Hong Kong SAR, China; 9 Gerald Choa Neuroscience Institute, The Chinese University of Hong Kong, Hong Kong SAR, China

**Keywords:** Depression, Psychiatric disorders

## Abstract

The omega sign is a melancholic facial expression, resembling the Greek letter omega “Ω”. To study static omega sign (visible full-blown Ω-shaped or vertical wrinkles at rest) and dynamic omega sign (newly appearing or deepening) with digital assessment for depression via deep learning, subjects with major depressive disorder (MDD) and controls were invited to take neutral photos and videotaped interviews to study the static and dynamic omega sign, respectively. A deep residual learning model was used to generate automatic prediction. In total, 252 MDD subjects (age: 50.9 ± 11.1 years; 66.7% female) and 192 age- and sex- controlled subjects (age: 49.6 ± 11.7 y; 62.0% female) were recruited. The presence of static omega sign was marginally associated with an increased risk for MDD (MDD subjects: 24.3%, Controls: 16.0%; OR = 1.79, 95% CI = 0.99 to 3.44, *P* = 0.06). In particular, comorbid MDD and generalized anxiety disorder (GAD) subjects had a significantly higher rate (45.5%) than controls (OR = 6.36, 95% CI = 1.71 to 23.63, *P* = 0.006). The frequency of dynamic sign was associated with the presence of current moderate to severe depression (OR = 1.04, 95% CI = 1.01 to 1.07, *P* = 0.02). In summary, MDD patients, especially comorbid with GAD, tended to have a higher rate of static omega sign than controls. The frequency of dynamic sign was associated with an increased risk of having moderate to severe depression. The advance of digital-AI technology offers a novel strategy to provide objective measurement of the omega sign that will assist clinical assessment of depression.

## Introduction

The omega sign on the forehead was first described by Charles Darwin in 1872 as a facial expression of grief and anxiety [1]. It comprises of central skin furrow (caused by inner brow raising) and vertical furrows (due to eyebrows narrowing), resembling the Greek letter omega “Ω”. A group of facial musculatures are involved, including the medial part of the frontalis for inner brow raising, corrugator supercilii and the medial orbicularis oculi for eyebrows narrowing. The latter two muscles often act together with the procerus and the depressor supercilii, resulting in simultaneous depression of eyebrows [2]. The omega sign described by Darwin was characterized by dynamic changes in facial expressions, specifically with the newly appearing or deepening of wrinkles under stressful conditions [3]. Thus, the dynamic omega sign probably reflects the psychomotor expression of the instant emotional state in the facial musculature [4]. After years of repeated muscle contraction together with a decline in skin elasticity, the persistent wrinkles may be visible even at rest resulting in static omega sign [5, 6]. The natural progression of the omega sign was postulated to start with vertical wrinkles between eyebrows followed by the full-blown omega pattern [7].

Several researchers have applied electromyographic (EMG) measurement to investigate the neurophysiology of the omega sign. In 1959, Whatmore et al. [8] found that depressed patients, especially those with psychomotor retardation, had a significantly higher residual motor activity in multiple regions (including frontalis and corrugator muscles) than controls. Around 1980s, Greden et al. [9] reported that endogenously depressed patients had greater corrugator activity levels than non-endogenously depressed patients during happy and sad imagery. Schwartz et al. [10] showed that nondepressed subjects tended to have a greater decrease in corrugator activity levels during happy imagery, and a greater increase in frontalis during sad imagery. Nonetheless, there was no difference of corrugator activity during happy imagery between depressed and nondepressed group in hospitalized psychiatric patients [11]. Besides, a significant positive correlation was found between agitation and corrugator activity during resting, happy, and typical day imagery tasks among women with depression [12].

However, the interest in this facial omega sign as behavioral insight into depression quickly faded [13]. There have been only isolated case reports [14, 15] that re-emphasized its importance in the assessment of depression. Current gold standard for the diagnosis of depression relies on clinical interview, which serves as a major barrier for timely assessment of a large number of patients [16]. Besides, conventional clinical approaches, such as interviews and questionnaires, largely rely on patient self-reporting. This often provides an incomplete understanding of the patient’s condition [17], and some patients may be too unwell to participate reliably. Thus, objective measurements are needed to alleviate the burden of health care services, and aid clinicians to capture symptoms and signs more accurately and continuously. In this context, the omega sign, a classical and easily identifiable facial sign on the forehead that would not be covered by the mask (even during the pandemic situation), may provide added diagnostic value for depression, making it an important subject for further research and application.

Nevertheless, the objective measurement of the omega sign in routine clinical practice is still rare. Facial EMG is inconvenient and inaccessible in most daily clinical settings, and is limited by several technical concerns including inter-operator variability and muscle crosstalk [18]. The exact rate and frequency of static and dynamic omega sign and its clinical correlates with depression remains unclear, albeit it has been postulated to be higher in depression as related to the frequent distress associated with repeated eyebrow movement and skin pathologies contributed by brain-skin circuit [6, 19]. The advance of digital technology (e.g. digital measurement with deep learning) [20] will be a promising strategy to provide accessible, reliable and accurate assessment of clinical signs and symptoms. Thus, the study aimed to investigate the static and dynamic omega sign among depressive patients and controls with digital assessment and deep learning methods.

## Methods

### Subject recruitment

This was a case-control study conducted between October 2020 and July 2023 as part of an ongoing project on the development of digital phenotyping in depression [21]. Patients with lifetime major depressive disorder (MDD) between 18 and 65, and age- and sex-matched controls free from psychiatric disorders were recruited. The majority of the MDD subjects (82.1%) were recruited with a convenient sampling from a public regional psychiatric clinic in Hong Kong and the rest of the subjects were recruited from the community. The diagnoses of all subjects were confirmed via the Structured Clinical Interview for DSM-5 – Clinician Version (SCID-5-CV) [22] by a trained medical researcher, at which the information on duration of illness, number of major depressive episodes (MDEs), and comorbidities were documented. The controls were recruited from both community and sleep centre of the psychiatric department and were ascertained as free from lifetime psychiatric disorders by SCID-5-CV. The exclusion criteria for all study subjects included: 1) lifetime history of bipolar disorder, schizophrenic-spectrum disorder, dementia, intellectual disability, and neurological disorders; 2) presence of clear confounding factors (e.g., facial injury, speech disorder, or history of Botox facial injections); and 3) invalid photos and videos due to technical issues (e.g., forehead covered by hair or device battery depletion). The study was approved by the Joint Chinese University of Hong Kong – New Territories East Cluster Clinical Research Ethics Committee (The joint CUHK–NTEC CREC, Ref No: 2020.492).

### Clinical assessment

In addition to clinical interview, the Structured Interview Guide for the Hamilton Depression Rating Scale with Atypical Depression Supplement (SIGH-ADS) [23] was administered for assessing the severity and symptoms of depression. The presence of current moderate to severe depression in MDD subjects was defined as HAMD-17 score ≥ 14 [24]. Anxious depression was defined as MDD with HAMD-17 Anxiety/Somatization factor score ≥ 7 [25, 26]. Details of sociodemographic features (age, sex, marital status, education level, and family income) and current consumption of tobacco and alcohol were collected via self-report questionnaires. Information on current psychotropic use was obtained from electronic medical system [27, 28].

### Digital measurement

Neutral photos were collected by iPad Air 2 for studying static omega sign while dynamic omega signs during videotaped interview were captured by SONY HDR-CX405 Handycam. Subjects were instructed to relax and look at the iPad Air 2 and photos were taken with mask or without mask from a distance of approximately 60 cm. In addition, we also instructed them to frown for several seconds and took photos of their posed facial expressions. Neutral photos (relaxed) and the photos of posed facial expression were collected for training and validation of deep learning models. For videotaping, the duration of the interview normally took 15–30 min. All the photo-taking and videotaping were performed in clinical consultation rooms with usual room lighting.

### Deep learning-based omega sign detection

**Fig. 1:**
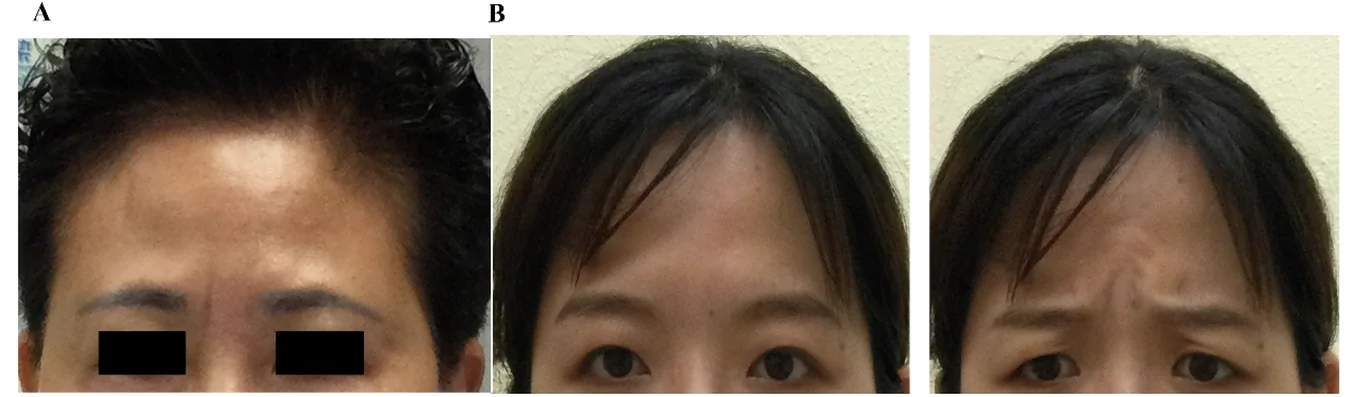
The examples of static and dynamic omega sign in this study. **A** Static omega sign. **B** Dynamic omega sign. Left was neutral photo and right was newly appearing full-blown omega pattern. The photos were taken and published with the written informed consent of the individuals.

In this study, static omega sign was defined as either full-blown Ω-shaped wrinkles or vertical wrinkles between eyebrows [7]. Dynamic omega sign was referred to newly appearing omega sign (including vertical wrinkles) or deepening of existing static sign. As our subjects seldom showed full blown Ω-shaped wrinkles which would predominantly occur during strong emotions (e.g., crying) [1, 29], the dynamic sign mostly appeared as vertical wrinkle(s) (Fig. 1).

#### Training and validation data

The data used for building a deep learning model consisted of Real-world Affective Faces Database (RAF-DB) [30] and photos from 64.8% of our subjects (*n* = 271). The remaining 35.2% of our local dataset (*n* = 147) were not annotated manually. RAF-DB is a large-scale facial expression database sourced from social networks, with large individual variation in age (0–70 years old), gender (52% female, 43% male, and 5% unsure), and race (77% Caucasian, 8% African American, and 15% Asian). An annotator (J.C.) performed the labelling of the photos into 3 categories (1 - presence of the omega sign, 0 - absence of the omega sign, and U - uncertain). Overall, 4883 photos (RAF-DB: 0: *n* = 3276, 1: *n* = 614; Our 271 subjects: 0: *n* = 454, 1: *n* = 539) were labelled manually for model training and validation. The mastery skill of the labelling of facial expression by J.C. as well as the process of face detection and alignment was described in Supplementary Methods.

#### Training and validation of classification models

A deep residual learning model called residual network (ResNet) [31] was used to generate automatic prediction. ResNet is a state-of-the-art method for various computer vision tasks like image classification, feature extraction, and image segmentation. It allows training on much deeper neural networks than the conventional Convolutional Neural Networks (CNNs), a classic artificial neural network for image data. It learns the residual functions with reference to the layer inputs, which is easier to optimize than the unreferenced underlying mapping of CNNs. The residual function is realized by “shortcut connections” within the feedforward neural networks, where it simply performs an identity mapping by skipping one or more layers in the network. In this regard, ResNet can overcome the degradation problem – higher training error when depth increases, for deeper neural networks. The ResNet with 18 layers (ResNet-18) was first pretrained using Imagenet [32], and then fine-tuned with all RAF-DB images and 90% of the images from our annotated subjects to obtain the base model for predicting the presence of the omega sign. The remaining 10% of our annotated images were used for validation. Given the limited number of samples, 90:10 split as the minimum split in common practice was used [33]. Both oversampling and undersampling methods were applied to ensure the training set achieving a balanced representation of each class. A 5-fold cross-validation using all RAF-DB and our images was also conducted to test the internal validity of the ResNet-18 model. Same resampling procedures were applied to the training set on each iteration. Further, by using 90% of the RAF-DB images for training and 10% of the RAF-DB images for validation to determine the best model, we subsequently used this model on our entire images for external validation. Finally, we performed transfer learning on the base model to generate an updated model that detected deepening of the omega sign. This was achieved by using 117 neutral photos with static sign and paired posed (deepening) photos.

### Application of classification model

The base model was adopted to estimate the rate of static sign in the neutral photos among our entire dataset, including both annotated and unannotated subjects. For capturing dynamic signs in videos, the base model was used for subjects without static wrinkles (newly appearing), while the updated model was selected for subjects with static wrinkles (deepening). More details about exploring potential mask effect on predicting static sign in photos, and application of the base model to videos were provided in Supplementary Methods. The frequency of dynamic sign per subject per frame during single interview was calculated as N / T (N: Number of frames in which dynamic sign was present; T: Total frames of the interview).

### Statistical analysis

Descriptive data was presented as mean ± standard deviation (SD) or frequency (%). Shapiro-Wilk test was applied for assessing normality. To investigate differences between two groups, Mann-Whitney U test was used for non-normally distributed continuous variables. As for intergroup differences of categorical variables in 2 × 2 contingency tables, Chi-square test was used when no cell had an expected count less than 1, and that no more than 20% of the cells had an expected count less than 5. Fisher’s exact test was chosen if these rules of thumb were violated. Binary and multinomial logistic regression analysis was adopted to study clinical correlation of static and dynamic sign (independent variable) while controlling for covariates. To further investigate whether the association of dynamic sign with depression was confounded by concurrent anxiety symptoms or anxiety disorders, binary logistic regression analysis was used among MDD subjects after adjustment for age, sex, and the severity of depression (excluding the items used to identify anxious depression). As for testing potential medication effect, adjustment was made for age and sex. Spearman correlation was applied to show the correlation between frequency of dynamic omega sign and HAMD-17 score. A two-tailed *p* value of < 0.05 was considered statistical significance. All data acquired was processed and analyzed using IBM SPSS Statistics 27.

## Results

### Demographic as well as alcohol and tobacco use information

**Fig. 2:**
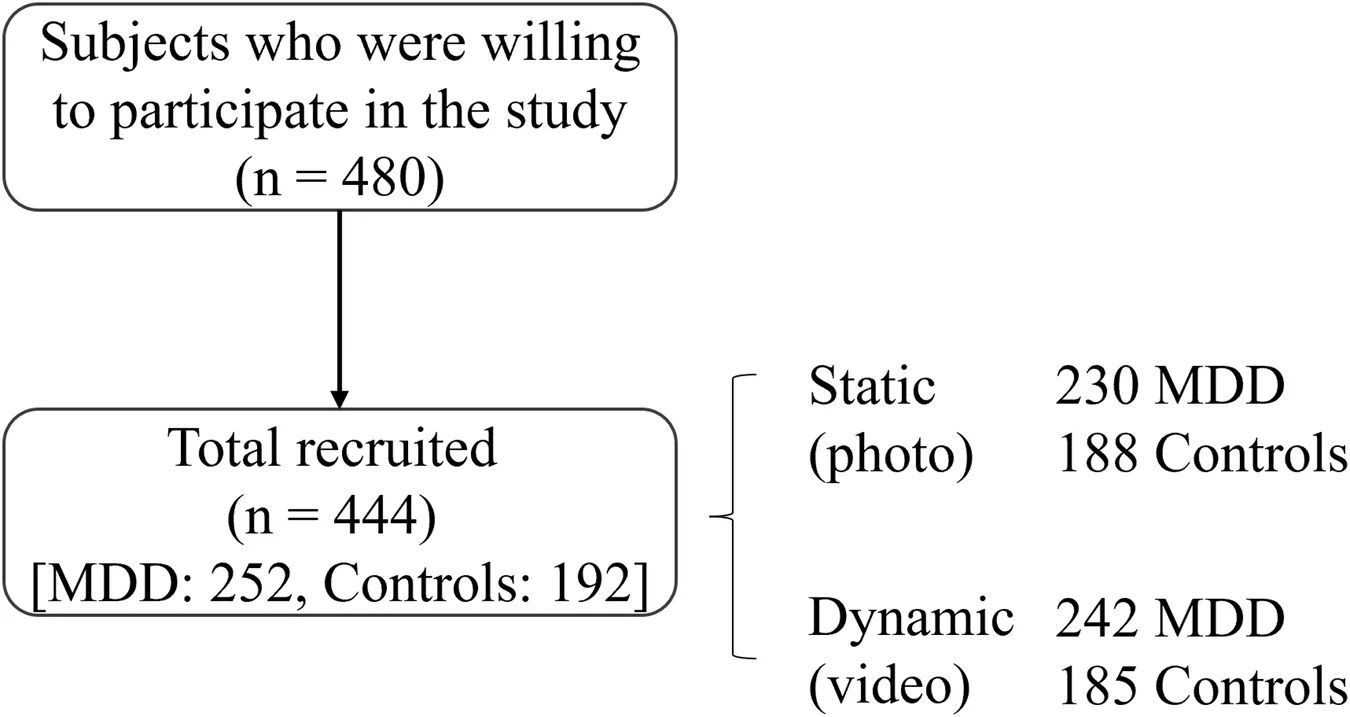
Flow diagram of the study. 252 MDD subjects and 192 controls were eligible for the final analysis. Among them, the neutral photos of 230 cases and 188 controls were taken (static omega sign study), and the clinical interviews of 242 cases and 185 controls were videotaped (dynamic omega sign study).

In total, 252 MDD subjects (age: 50.9 ± 11.1 years; 66.7% female) and 192 age- and sex- controlled controls (age: 49.6 ± 11.7 y; 62.0% female) were eligible for the final analysis. Among them, the neutral photos of 230 cases and 188 controls were taken (static omega sign study), and the clinical interviews of 242 cases and 185 controls were successfully videotaped (dynamic omega sign study). Figure 2 demonstrated the flow diagram. MDD subjects were more likely to be single, unemployed (*P* = 0.003), and have a lower education level (*P* < 0.001) and family income (*P* < 0.001). MDD subjects were more likely to smoke (*P* = 0.002), whereas alcohol assumption was comparable between two groups (Table 1).

**Table 1:** Sample characteristics.

	Controls (*n* = 192)	MDD subjects (*n* = 252)	Analysis		
	%	%	X^2^	Cramer’s V	P
Female	62.0	66.7	1.05	0.05	0.31
Marital status					
Married/Cohabitating	71.6	57.3	8.62	0.15	0.003^**^
Employment status, yes,	69.8	51.7	13.30	0.18	< 0.001^***^
Education					
Tertiary level or above	55.2	37.3	12.73	0.18	< 0.001^***^
Family income monthly < 15,000 HKD	11.5	35.4	29.53	0.28	< 0.001^***^
Smoking, occasionally or often	2.2	9.8	9.47	0.16	0.002^**^
Alcohol use, occasionally or often	16.9	13.5	0.88	0.05	0.35
Static omega sign	16.0	24.3	4.46	0.10	0.04*
	Mean (SD)	Mean (SD)	Z	r	P
Age, y	49.6 (11.7)	50.9 (11.1)	−1.08	−0.05	0.28
Frequency of dynamic omega sign, %,	7.9 (9.1)	9.3 (13.5)	−0.15	−0.007	0.88

Comparisons were conducted by using Mann-Whitney U test or Chi-square test where applicable. r = The Rank-Biserial Correlation Coefficient.

^***^P < 0.001, ^**^P < 0.01, ^*^P < 0.05.

### Evaluation of deep learning-based omega sign detection models

**Fig. 3:**
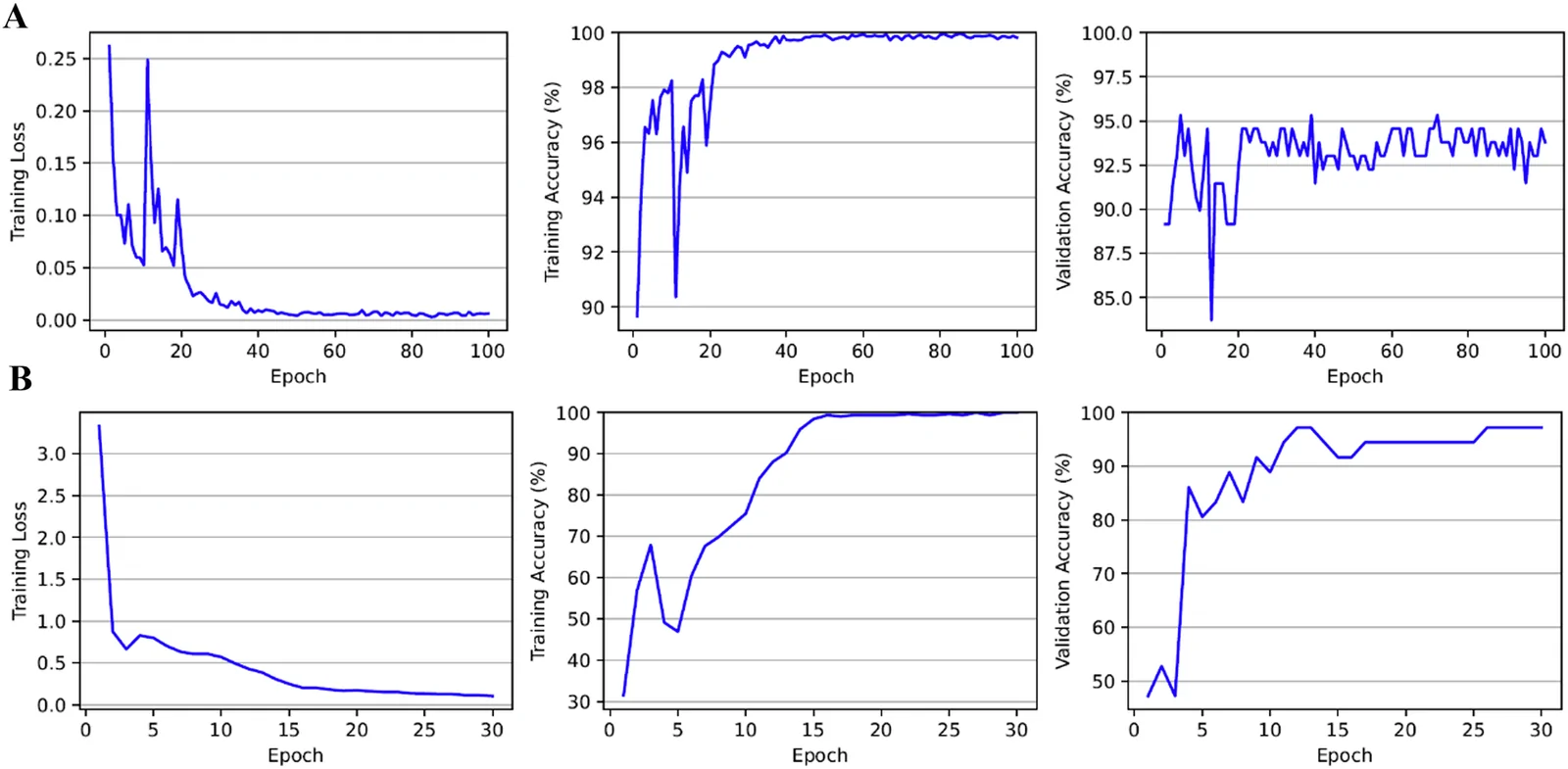
Prediction performance of the base model and updated model. **A** The process of fine-tuning the base model was achieved by 100-epoch training. The training process stopped at the 72nd epoch with the highest validation accuracy over 95%. **B** The process of fine-tuning the updated model was achieved by 30-epoch training. The training process stopped at the 30th epoch with highest validation accuracy over 97%.

The process of fine-tuning the base model that identified static omega sign and newly appearing dynamic omega sign was achieved by 100-epoch training. The training process stopped at the 72nd epoch with the highest accuracy over 95% achieved in the validation set as shown in Fig. 3. A separate 5-fold cross-validation was also conducted, and Supplementary Fig. 1 summarized the average accuracy (around 95%). For external validation, the model had an 85.5% accuracy on our independent local images. In terms of the updated model which captured deepening dynamic sign, the training stopped at the 30th epoch with the highest validation accuracy above 97% (Fig. 3). As for the assessment of mask effect on predicting static omega sign, the average validation accuracy was similar (95–96%) for photos with and without surgical mask (Supplementary Fig. 2).

### The rate of static omega sign

**Fig. 4:**
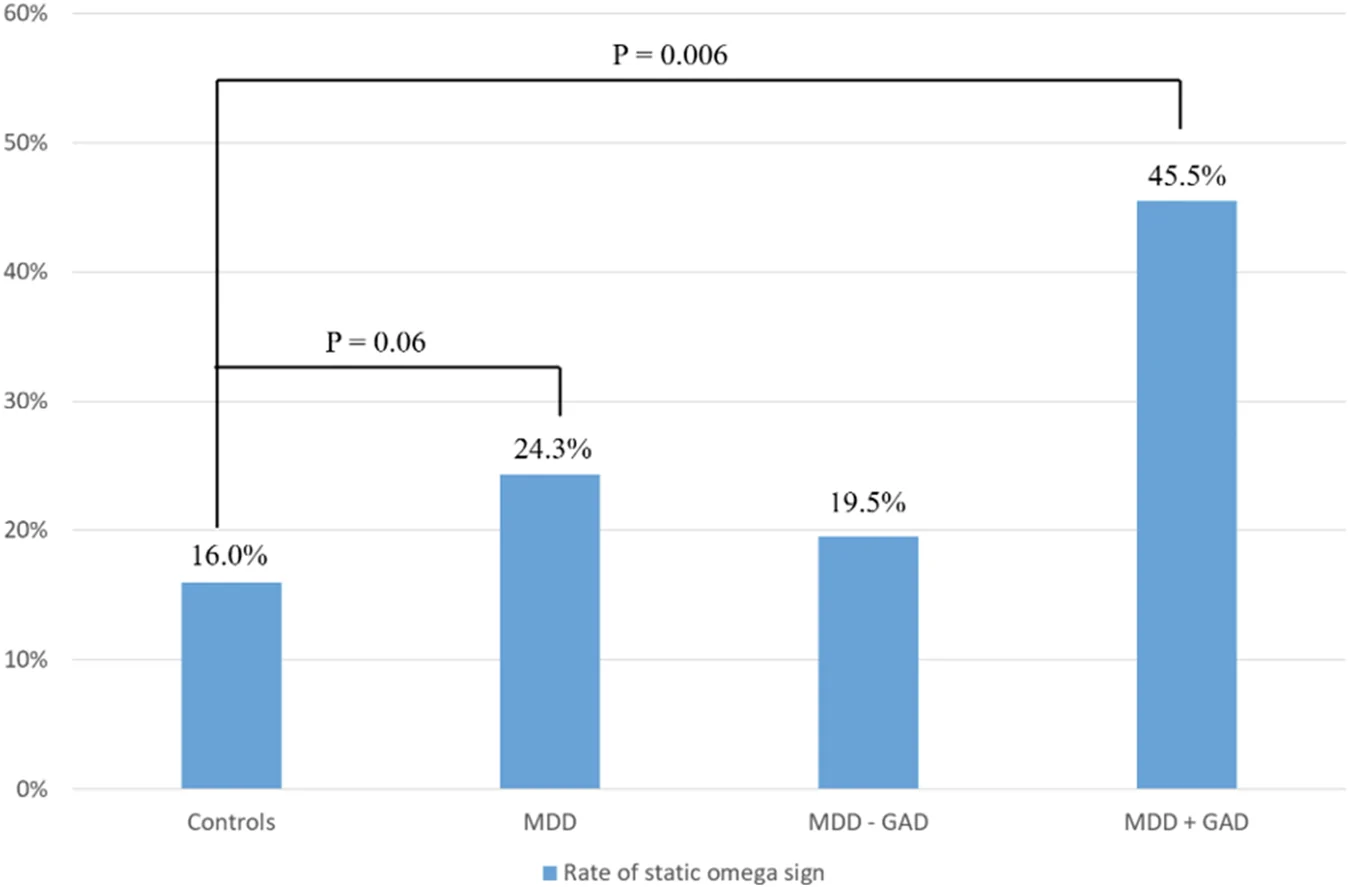
Column graph showing the rate of static omega sign (mean). Binary logistic regression analysis was used to compare controls and MDD subjects. Multinomial logistic regression analysis was adopted to compare controls, MDD – GAD subjects, and MDD + GAD subjects. All the analyses were adjusted for age, sex, marital status, employment status, education, family income, and smoking, with static omega sign treated as independent variable.

The rate of static omega sign labelled by automated model was significantly higher in MDD subjects (*n* = 230) than controls (*n* = 188) (24.3 vs. 16.0%, *P* = 0.04). (Table 1) After adjustment for age, sex, marital status, employment status, education, family income and smoking, static omega sign was marginally associated with an increased risk for MDD (OR = 1.84, 95% CI = 0.99 to 3.44, *P* = 0.06) (Fig. 4).

Among MDD subjects, static omega sign was more commonly seen in male subjects (*P* = 0.01), older age (*P* < 0.001) and those with comorbid generalized anxiety disorder (GAD) (*P* = 0.01). Other demographic and clinical variables including duration of depressive illness, history of multiple MDEs, current psychotropic usage and HAMD-17 score, and other comorbidities were not related to the presence of static omega sign (Table 2).

**Table 2:** Association of static omega sign with potential predictors among MDD subjects.

Variables	Static omega sign				
	No (174)	Yes (56)	Analysis		
	%	%	X^2^	Cramer’s V	P
Male	28.7	46.4	5.99	0.16	0.01^*^
Marital status					
Married/Cohabitating	40.4	39.1	0.02	0.01	0.88
Employment status, yes	53.8	41.3	2.18	0.11	0.14
Education					
Tertiary level or above	38.8	23.9	0.02	0.13	0.07
Family income monthly < 15,000 HKD	37.2	31.8	0.42	0.05	0.52
Smoking, occasionally or often	11.2	7.0	/	0.06	0.57
Alcohol use, occasionally or often	15.9	4.7	3.62	0.14	0.06
Number of major depressive episodes ≥ 2	48.6	47.5	0.02	0.01	0.90
Anxious depression	20.7	26.8	0.91	0.06	0.34
Psychotropic					
Antidepressants	66.3	79.2	3.20	0.12	0.07
Anxiolytics	30.2	26.4	0.28	0.04	0.60
Antipsychotics	20.1	26.4	0.94	0.07	0.33
Mood stabilizer	5.3	7.5	/	0.04	0.52
Polypharmacy (≥ 2 psychotropics)	36.7	39.6	0.15	0.03	0.70
Comorbidity					
Panic disorder (lifetime)	10.1	6.3	/	0.06	0.58
Agoraphobia (current)	5.3	4.2	/	0.02	1.00
Social phobia (current)	5.9	4.2	/	0.03	1.00
OCD (current)	2.4	0	/	0.07	0.58
GAD (current)	7.1	20.8	/	0.19	0.01^*^
Psychotic features (lifetime)	8.9	16.7	2.40	0.11	0.12
PTSD (current)	0.6	4.2	/	0.13	0.12
	Mean (SD)	Mean (SD)	Z	r	P
Age, y	49.5 (11.3)	57.9 (6.9)	−5.29	−0.35	< 0.001***
HAMD-17 score	9.0 (6.4)	10.5 (7.4)	−1.21	−0.08	0.23
Duration of illness, y	14.4 (10.9)	14.8 (11.0)	−0.23	−0.02	0.82

*OCD* obsessive-compulsive disorder, *GAD* generalized anxiety disorder, *PTSD* post-traumatic stress disorder. Comparisons were conducted by using Mann-Whitney U test, Chi-square test, or Fisher’s exact test where applicable. r = The Rank-Biserial Correlation Coefficient.

***P < 0.001, *P < 0.05.

We further divided MDD subjects into MDD - (without) GAD (*n* = 195) and MDD + (with) GAD (*n* = 22) groups. The rate of static omega sign was 19.5% in MDD - GAD group and 45.5% in MDD + GAD group respectively. After adjustment for covariates (age, sex, marital status, employment status, education, family income and smoking), static omega sign was significantly associated with an increased risk for MDD + GAD (OR = 6.36, 95% CI = 1.71 to 23.63, *P* = 0.006), but not for MDD - GAD (*P* = 0.20) (Fig. 4).

When comparison was made between MDD subjects from psychiatric clinic and MDD subjects from community (Supplementary Table 1), the clinic subjects showed higher severities in various depressive features (HAMD-17 total score, *P* = 0.03; duration of illness, *P* = 0.02; history of recurrent MDEs, *P* = 0.02; anxious depression: *P* = 0.02; psychotic features: *P* = 0.008), and were poorer (*P* = 0.04) and less likely to be employed (*P* = 0.01). Patients recruited from the clinic had an apparently higher rate of static sign than those from community (26.1 vs. 16.7%), but the difference did not reach statistical significance (*P* = 0.20).

### The frequency of dynamic omega sign

**Fig. 5:**
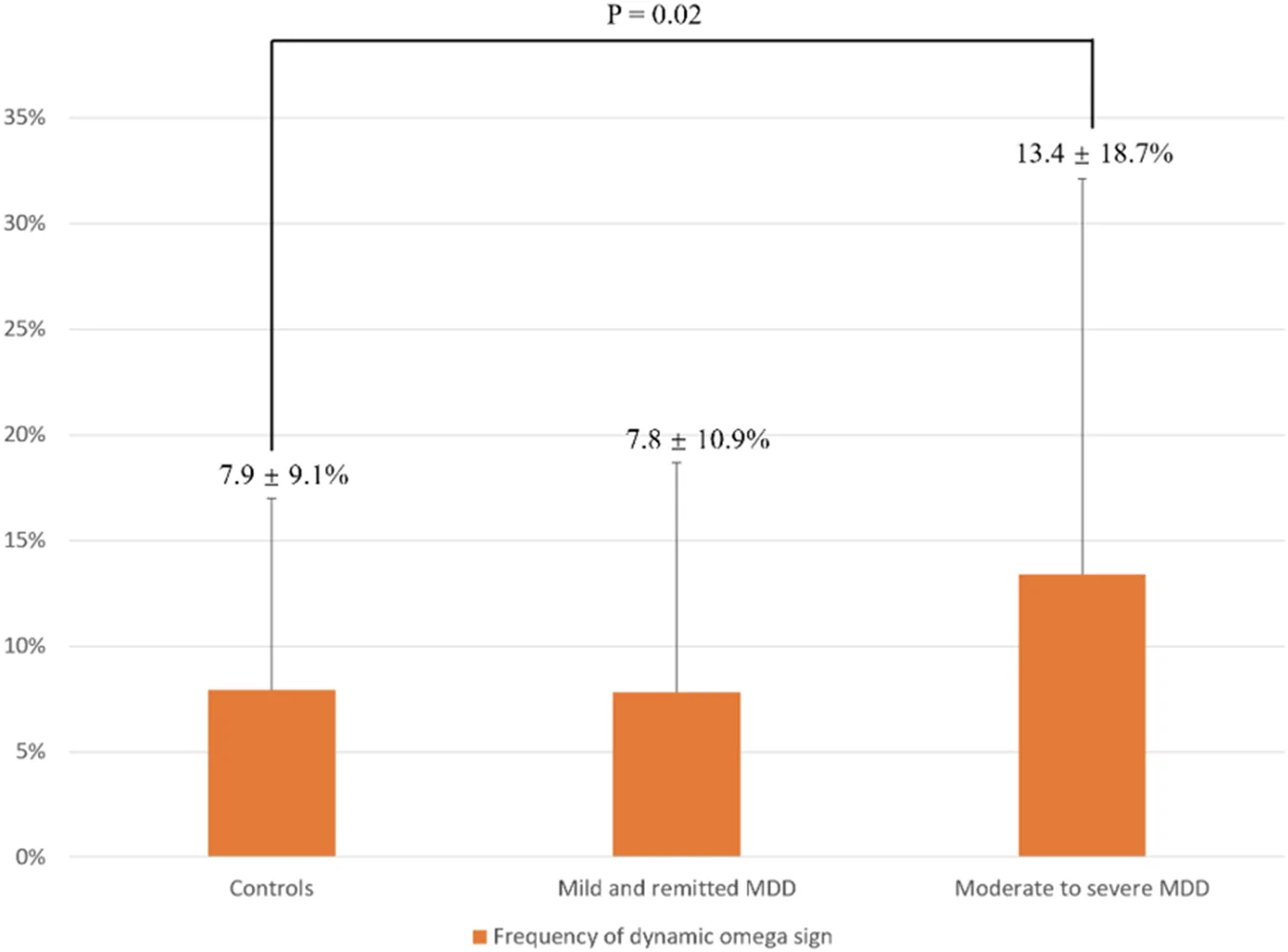
Column graph with error bars showing the frequency of dynamic omega sign (mean and standard deviation). Multinomial logistic regression analysis was adopted to study clinical correlation of dynamic omega sign (independent variable) while controlling for age, sex, marital status, employment status, education, family income and smoking.

The frequency of dynamic omega sign was comparable between MDD group (*n* = 242) and controls (*n* = 185) (MDD: 9.3 ± 13.5% vs. Controls: 7.9 ± 9.1%, *P* = 0.88). (Table 1) No difference of frequency of dynamic sign was found between MDD patients from clinic (*n* = 22) and those from community (*n* = 22) (*P* = 0.13). (Supplementary Table 1) Among MDD subjects, results from Spearman correlation showed that the frequency of dynamic omega sign was correlated with HAMD-17 score (r = 0.13, *P* = 0.046). The frequency of dynamic omega sign was significantly higher in moderate to severe MDD (*n* = 63), when compared to mild and remitted MDD group (*n* = 179) (13.4 ± 18.7% vs. 7.8 ± 10.9%, *P* = 0.03). After adjustment for age, sex, marital status, employment status, education, family income and smoking, moderate to severe MDD subjects were more likely to demonstrate dynamic omega sign than controls (OR = 1.04, 95% CI = 1.01 to 1.07, *P* = 0.02) (Fig. 5).

To investigate whether this association could be accounted by concurrent anxiety symptoms or anxiety disorders, we further tested the association of dynamic sign with anxious depression or anxiety spectrum disorders among MDD subjects. There was no significant association of dynamic sign with anxious depression (*P* = 0.48) or any comorbid anxiety spectrum disorder (Panic disorder: *P* = 0.38; Agoraphobia: *P* = 0.22; Social phobia: *P* = 0.31; Obsessive-compulsive disorder (OCD): *P* = 0.82; GAD: *P* = 0.29; Post-traumatic stress disorder (PTSD): *P* = 0.24) after adjustment for age, sex, and the severity of depression (excluding the items used to identify anxious depression). Among different psychotropics, the frequency of dynamic sign was only associated with the use of mood stabilizer (*P* = 0.045), but the use of mood stabilizer was not related to the severity of depression (*P* = 0.72) after adjustment for age and sex among MDD subjects.

## Discussion

To our best knowledge, this is the first study that provides data of the rate of static omega sign and frequency of dynamic omega signs among MDD and non-psychiatric controls by using digital assessment coupling with deep learning. Our data showed that static omega sign was marginally associated with an increased risk for MDD (MDD subjects: 24.3%, Controls: 16.0%; OR = 1.79, *P* = 0.06). In particular, subjects with comorbid MDD and GAD had a significantly higher rate (45.5%) than controls (OR = 6.36, *P* = 0.006). The frequency of dynamic sign was associated with the presence of a current moderate to severe episode of depression when compared to controls (OR = 1.04, *P* = 0.02).

A slightly higher rate of static sign in MDD subjects was consistent with our hypothesis, probably due to their repeated eyebrow movement and brain-skin circuit related skin pathologies [19]. Mental stress not only initiates nerve-regulated facial muscle contraction [34], but may also fasten skin aging process via complex neuroendocrine pathway. Various hormones, neurotransmitters, neuropeptides, and neurotrophins have been reported as stress mediators that could trigger inflammation and allergy in skin [35]. Notably, nearly one in 2 subjects (45.5%) with comorbid MDD and GAD had static omega sign. Experimental studies have shown that this specific facial frowning movement was frequently seen in individuals who were engaging in mental [36] or physical [37] effort tasks. This may also explain a relatively high rate of static sign in controls (about 1 in 6 subjects), suggesting repeated forehead muscle contraction is relatively common even in normal subjects. Future studies are warranted to test the specificity of static omega sign with larger sample size of depressed, other psychiatric and non-psychiatric populations. Repeated muscle movements leading to the appearance of static sign might probably be non-specific to depressed mood and often involved in various situations (e.g. anxious) when hypervigilance is needed. Besides, the comorbidity of MDD and GAD might indicate increased chronicity and psychosocial disability [38, 39]. Although we failed to find any association of static sign with duration or recurrent episodes of MDD, other information such as total duration of episode(s) and adverse life events might be more reflective of accumulative stress.

The frequency of dynamic omega sign was associated with the presence of a current moderate to severe episode of MDD in our study, independently of anxiety and medications. The dynamic sign as reflected by the vertical furrows was mainly related to corrugator muscle activity. Paul Ekman similarly described and named the facial expression of brow lowering (Action unit, AU 4) that was caused by the corrugator muscle movement [5]. AU 4 was proposed to be involved in emotions including sadness, anger, anxious, confusion and fear [40, 41], which were commonly seen in depressed patients. Interestingly and consistently, more brow lowering (AU 4) facial expression during ecological momentary assessment with our self-developed app (D-MOMO), in combination with other multimodal digital assessment, predicted the presence of a lifetime history and non-remission status of MDD [21].

The findings of current study have several implications. We showed that both static and dynamic omega sign have the potential to serve as candidate markers of depression, which has been preliminarily supported by our recent multimodal digital assessment of depression [21]. Additionally, the use of accessible digital device coupling with deep learning analysis provides more reliable results than eyeballing that may be subjective to visual error [42]. Comparing to the sophisticated EMG measures or the crude visual examination, the digital-artificial intelligence (AI) system may offer the advantage of easy and reliable phenotyping of MDD based on the omega sign, even with mask-wearing conditions during the pandemic situation. Besides, facial feedback hypothesis suggests that facial expressions function not only as external displays but also as internal modifiers of emotion [43]. Consistently, injection of botulinum toxin into the glabellar region of the face has been suggested as a novel and effective therapeutic approach in the treatment of depression [44]. Our findings added further evidence and suggested the need for further understanding of the complex interaction of facial expression and mood features in depression, across neuroscientific, digital and therapeutic perspectives.

There were some limitations in this study. First, the researcher who participated in human labeling of static omega sign and clinical rating of depressive symptoms was not blind to the diagnosis of subjects. This may lead to potential rater bias even with the use of automatic prediction. Nevertheless, the automated model achieved high validation accuracy which suggested a minimal bias. Second, despite good validation accuracy for both base and updated model, data for model training was relatively modest and primarily based on multi-ethnic photos. A relatively lower but still robust external validation performance suggested that there may be subtle cross-cultural or cross-ethnic variability in facial expression. Thus, the necessity of larger, multicentre, and cross-cultural external validation datasets should be emphasized to ensure the robustness and generalizability of the model. As for future refinement of our automated models (ideally into one model), more training data is necessary. Third, the use of convenience sampling in the study might introduce selection bias and limited generalizability of our results. Fourth, the selection of automated models in video analysis was manually determined in this study. Future fully automated model will be needed. Fifth, despite the lack of significant difference of the rate of static omega sign and the frequency of dynamic omega sign between clinic-recruited and community-recruited MDD subjects, clinic-recruited subjects were relatively more severe, poorer, and unemployed. The heterogeneity of these two sources of depressed subjects regarding the omega sign should be re-evaluated with larger samples, particularly the community-recruited MDD subjects.

In summary, MDD patients, especially comorbid with GAD, tended to have a higher rate of static omega sign than controls. The frequency of dynamic sign was associated with current moderate to severe depression. The automated model with use of deep learning method in the identification of the omega sign would have the potential in assisting regular clinical care of depression.

## Supplementary information

Supplementary Methods, Supplementary Figures, Supplementary Table

## Data Availability

The data that support the findings of this study are available from the corresponding author upon reasonable request.
