# Supplementary material for: Capturing omega sign in the clinical assessment of depression by deep learning
Source: Transl Psychiatry. 2026 Jul 7;16:499. doi: 10.1038/s41398-026-04245-6 (PMC13623902; doi:10.1038/s41398-026-04245-6)
Supplement: Supplementary file 1 [file 41398_2026_4245_MOESM1_ESM.docx]

**Supplementary Methods**

*Training and validation data*

Reliability of the annotator

Inter-rater reliability of the omega sign scoring was conducted among a panel of investigators (n = 6) with 400 randomly selected photos of the subjects (Intraclass Correlation Coefficient was 0.96 based on average measures, absolute-agreement, 2-way mixed-effects model), which supported the reliability of the manual labelling. J. C. (first author) has obtained the certification from Paul Ekman Group after completing the Facial Action Coding System Manual training, as a proof of mastering in identifying all visually discernible facial movements.

Face detection and alignment

We applied a deep learning-based method called Multi-task Cascaded Convolutional Networks (MTCNN)(1) for face detection and alignment in Python. The framework exploited the inherent correlation between the two tasks by jointly learning the bounding box of the face and the locations of facial landmarks, and could handle various poses, illuminations, and occlusions of the image.

*Application of classification models to photos and videos*

Mask effect on predicting static omega sign

A 5-fold cross-validation was used to train binary classification models on four-fifths of 164 photos without mask (0: n = 92, 1: n = 72, according to human labelling) using ResNet-18, and then the performance of the trained models was evaluated on the rest one-fifth of photos and their paired photos with artificial surgical mask (image of a typical surgical mask cut out via Photoshop) on each iteration respectively. Finally, the average validation accuracy for 5-fold cross-validation was calculated.

Application of the base model to videos

Because the digital device used was different from the one in photo analysis, the presence of static sign in video analysis was determined by J. C. after viewing several randomly selected frames without visually discernible facial movements. Ultimately, 95.2% (80/84) of subjects with static omega sign in neutral photo identified by the base model were determined as having static sign in video analysis.

**References**

1.	Zhang K, Zhang Z, Li Z, Qiao Y. Joint Face Detection and Alignment Using Multitask Cascaded Convolutional Networks. IEEE Signal Processing Letters. 2016;23(10):1499-503.

**Supplementary Figures**

**Supplementary Figure 1** 5-fold cross-validation performance of the base model

This figure showed average accuracy (around 95%) of the base model over the 5 iterations.

**Supplementary Figure 2** Mask effect on predicting static omega sign

A 5-fold cross-validation was used to train binary classification models on four of five folds of photos without mask. Then, the average performance of the trained models was evaluated on the rest fold of photos, and their paired photos with artificial surgical mask respectively. The average validation accuracy was similar (95% - 96%) for photos with and without artificial surgical mask.

**Supplementary Table**

**Supplementary Table 1** Characteristics of MDD subjects from different sources

|  | From psychiatric clinic  (n = 207) | From community  (n = 45) | Analysis | | |
| --- | --- | --- | --- | --- | --- |
|  | % | % | X^2^ | Cramer’s V | P |
| Female | 65.7 | 71.1 | 0.49 | 0.04 | 0.49 |
| Marital status |  |  |  |  |  |
| Married/Cohabitating | 57.1 | 58.5 | 0.03 | 0.01 | 0.86 |
| Employment status, yes | 47.3 | 70.0 | 6.65 | 0.18 | 0.01^*^ |
| Education |  |  |  |  |  |
| Tertiary level or above | 35.1 | 46.3 | 1.79 | 0.09 | 0.18 |
| Family income monthly < 15,000 HKD | 38.8 | 21.1 | 4.21 | 0.15 | 0.04^*^ |
| Smoking, occasionally or often | 11.0 | 4.9 | / | 0.08 | 0.38 |
| Alcohol use, occasionally or often | 13.3 | 14.6 | 0.05 | 0.02 | 0.82 |
| MDE ≥ 2 | 55.6 | 34.2 | 5.64 | 0.17 | 0.02^*^ |
| Anxious depression | 25.6 | 8.9 | 5.90 | 0.15 | 0.02^*^ |
| Comorbidity |  |  |  |  |  |
| Panic disorder (lifetime) | 10.4 | 6.8 | / | 0.05 | 0.58 |
| Agoraphobia (current) | 5.7 | 2.3 | / | 0.06 | 0.70 |
| Social phobia (current) | 5.7 | 2.3 | / | 0.06 | 0.70 |
| OCD (current) | 2.6 | 2.3 | / | 0.01 | 1.00 |
| GAD (current) | 11.4 | 2.3 | / | 0.12 | 0.09 |
| Psychotic features (lifetime) | 14.0 | 0 | 6.95 | 0.17 | 0.008^**^ |
| PTSD (current) | 2.1 | 0 | / | 0.06 | 1.00 |
| Static omega sign | 26.1 | 16.7 | 1.65 | 0.09 | 0.20 |
|  | Mean (SD) | Mean (SD) | Z | r | P |
| Age, y | 51.2 (11.1) | 49.4 (11.5) | -0.95 | -0.06 | 0.34 |
| HAMD-17 score | 9.9 (7.0) | 7.3 (5.5) | -2.21 | -0.14 | 0.03^*^ |
| Length of illness, y | 15.0 (10.6) | 11.5 (11.0) | -2.30 | -0.14 | 0.02^*^ |
| Frequency of dynamic omega sign, % | 9.2 (14.2) | 9.4 (9.9) | -1.52 | -0.10 | 0.13 |

OCD = obsessive-compulsive disorder; GAD = generalized anxiety disorder; PTSD = post-traumatic stress disorder. Comparisons were conducted by using Mann-Whitney U test, Chi-square test, or Fisher's exact test where applicable. r = The Rank-Biserial Correlation Coefficient. ^***^P < 0.001, ^**^P < 0.01, ^*^P < 0.05
